# Antimicrobial susceptibility of *Escherichia coli* isolated from diabetic patients in Mogadishu, Somalia

**DOI:** 10.3389/fmicb.2023.1204052

**Published:** 2023-08-24

**Authors:** Shafie Abdulkadir Hassan, Yousif Mousa Alobaid Ahmed, Gallad Dahir Hassan

**Affiliations:** ^1^Department of Medical Laboratory Sciences, Faculty of Medicine and Health Sciences, Jamhuriya University of Science and Technology, Mogadishu, Somalia; ^2^Department of Microbiology, Faculty of Medical Laboratory Sciences, University of El Imam El Mahdi, Kosti, Sudan; ^3^Department of Public Health, Faculty of Medicine and Health Sciences, Jamhuriya University of Science and Technology, Mogadishu, Somalia; ^4^School of Public Health and Research, Somali National University, Mogadishu, Somalia

**Keywords:** UTI, diabetes mellitus, *Escherichia coli*, antibiotic resistance, Somalia

## Abstract

**Background:**

Urinary tract infections (UTIs) are a major concern for diabetic patients due to the impact of diabetes on the urinary tract and immune system. *Escherichia coli* is the most common pathogen causing UTIs in diabetic patients and is known for its resistance. This study aimed to assess the antimicrobial susceptibility of *Escherichia coli* strains isolated from diabetic patients in Mogadishu, Somalia.

**Methods:**

This descriptive cross-sectional study was conducted at Ummah Hospital in Mogadishu, Somalia, from November 2021 to April 2022. Clean catch mid-stream urine specimens were collected from each participant and uropathogens were identified using standard techniques. The samples were cultured on CLED agar and antibiotic susceptibility patterns were determined using the Kirby-Bauer disk diffusion method. Data analysis was performed using SPSS version 20.

**Results:**

The overall prevalence of uropathogens among diabetic patients was 236/350 (67.5%) with *Escherichia coli* being the most common organism. According to multivariate logistic regression, the results showed that Female diabetic patients had a significantly higher likelihood of developing UTIs compared to males (AOR = 2.5, 95% CI: 1.357–4.522, *p* = 0.003). The duration of diabetes, history of previous UTIs, and previous antibiotic use were also significantly associated with UTIs. All isolates were found to be resistant to Cefotaxime (100%). In addition, high resistance rates were observed with ofloxacin (91%), ciprofloxacin (77.8%), amikacin (60.9%), ceftriaxone (58.3%), and cefepime (51.8%). The most sensitive antibiotics were colistin and imipenem (99.6 and 88.6%, respectively), followed by gentamycin (70%).

**Conclusion:**

This study found a high prevalence of uropathogens and increased multi-drug resistance. Continuous surveillance is needed to monitor uropathogen prevalence and resistance rates, guiding treatment recommendations, rational prescription programs, and policy decisions.

## Introduction

The prevalence of diabetes is increasing globally (Saeedi et al., 2019), and it is a major public health concern in many countries, including Somalia (Gele et al., 2016; Abdul et al., 2020). In 2019, it was estimated that 463 million individuals worldwide had diabetes mellitus, with type II DM accounting for 90% of cases, and in 2030, this number is predicted to increase to 552 million (Saeedi et al., 2019). Type 2 diabetes is a chronic, common metabolic disorder resulting in loss of or resistance to insulin which regulates blood glucose levels (Salari et al., 2022).

Patients with diabetes have a higher risk to acquire infections, especially occurring occur in the urinary tract (Mama et al., 2019). Moreover, diabetes can alter the host defense system in a number of ways, including impairing neutrophil function, increasing bacterial adhesion to the uroepithelial cells of diabetic individuals, and decreasing antibacterial activity due to glycosuria (Mohanty et al., 2022). Due to this, diabetic patients are more likely to have bacteriuria and complications as such abscesses, renal papillary necrosis, pyelonephritis, and emphysematous cystitis (Nitzan et al., 2015).

*Escherichia coli* is one of the most common bacterial pathogens associated with urinary tract infections in these patients (Ahmadishooli et al., 2020). Antibiotic resistance in *E. coli* is a serious problem, especially for diabetic patients (Wang et al., 2013; Sonkoue Lambou et al., 2022). The overuse and misuse of antibiotics have led to the development of antibiotic-resistant strains of *E. coli*, making it difficult to treat infections, due to the country’s ongoing conflict and limited healthcare resources, access to appropriate antibiotics is often limited (Aden and Bashiru, 2022). This has led to the widespread use of broad-spectrum antibiotics, contributing to the emergence and spread of resistant bacteria.

This study aimed to determine the antimicrobial susceptibility of *Escherichia coli* isolated from diabetic patients in Mogadishu, Somalia. By assessing the susceptibility patterns of these bacterial strains, we can gain valuable insights into the burden of antibiotic resistance in this population. Such information can help guide the development of appropriate treatment strategies and inform public health policy decisions.

## Methods

### Study design and area

This descriptive cross-sectional study was conducted at Ummah Hospital, Mogadishu, Somalia, from November 2021 to April 2022. Patients with one or more of the following symptoms; dysuria, frequency, urgency, Suprapubic discomfort, or flank pain, who have a clinical diagnosis were included in the study. Pregnant and non-diabetics were disqualified from the study. A total of 350 diabetic individuals were included in the study, including 115 men and 235 women who ranged in age from 10 to 80. Prior to collecting urine samples, all patients were informed of the purpose of the study and given the chance to provide their own consent.

### Sample collection and processing

Each patient was instructed to fill a sterile urine container with 10 to 20 mL of midstream urine. Within 1 hour of sample collection, samples were processed in the laboratory following proper guidelines to prevent contamination.

### Data collection

Demographic data and other information including clinical symptoms, previous antibiotics, and length of antibiotic usage were collected using a standardized questionnaire. Each patient enrolled in this study provided written consent.

### Isolation and identification of *Escherichia coli*

Using a calibrated loop (0.001 mL), urine samples were inoculated on cysteine-Lactose electrolyte deficient (CLED) medium plates to do urine cultures in a semi-quantitative technique. The plates were then incubated at 37°C for 18 to 24 h. Colony counts greater than 105 CFU/mL indicate significant bacteriuria and infection. Isolated colonies from significant plates were identified and differentiated from related organisms using standard conventional biochemical tests such as Kligler Iron, Indole, Urease, and Citrate test.

### Antimicrobial susceptibility testing

All isolates were tested for antimicrobial sensitivity using the Kirby-Bauer disk diffusion method and Colistin Broth Disk Elution test for colistin susceptibility, in accordance with the definition provided by the Committee of Clinical Laboratory International Standards (CLSI, 2020) on diagnostic sensitivity test plates. The McFarland standard was used to prepare bacterial inoculum suspensions for antimicrobial susceptibility testing. Mueller Hinton agar plates were streaked using a sterilized cotton swab (Gajic et al., 2022). Colistin susceptibility was assessed using the Colistin Broth Disk Elution test. Four tubes were prepared, each containing a different concentration of colistin: 0 μg/mL (growth control), 1 μg/mL, 2 μg/mL, and 4 μg/mL. To achieve these concentrations, 10 μg colistin disks were added to 10 mL of Cation-Adjusted Mueller Hinton broth, with the number of disks adjusted accordingly (0, 1, 2, or 4 disks). The tubes were then incubated at 35°C for 16–20 h. After incubation (16–20 h) at 35°C the colistin MIC values were read by visual inspection. MIC values of ≤1–2 μg/mL were considered susceptible, while MIC values ranging from 4 to >32 μg/mL were considered resistant to polymyxins.

The following concentrations of filter paper disks containing the antimicrobial medications were purchased from Hi-Media Laboratories: Amikacin (30 μg), Gentamycin (10 μg), Cefotaxime (30 μg), Meropenem (10 μg), Ciprofloxacin (5 μg), Ofloxacin (5 μg), Colistin (10 μg), and Cefepime (30 μg). To ensure the accuracy of antimicrobial susceptibility testing, reference strains of *E. coli* ATCC 25922 were employed as a means of quality control. The diameters of the zone of inhibition were interpreted based on CLSI guidelines.

## Results

In this study, we recruited a total of 350 patients with diabetes mellitus (DM), of which 26 (7.5%) had type I DM and 324 (92.5%) had type II DM. The majority of the participants were female, with 211 (60.3%) being female and 139 (39.7%) being male. 152 (43.5%) participants had symptoms suggestive of urinary tract infection (UTI). The overall prevalence of UTI among the diabetic population was 67.5%. The highest number of patients with urinary tract infections were in the age group of 50–60 years, with 87 (36.8%) individuals falling into this category.

### The factors associated with urinary tract infection

Risk factors were analyzed using logistic regression, including both univariate and multivariate analysis. The results indicated that female diabetic patients had a significantly higher likelihood of developing UTI compared to males (adjusted odds ratio [AOR] = 2.5, 95% confidence interval [CI]: 1.357–4.522, *p* = 0.003). Type 2 diabetes patients had a higher likelihood of UTI compared to type 1 diabetes patients, although the association was not statistically significant (AOR = 0.582, 95% CI: 0.234–1.450). The duration of diabetes has been found to have a significant association with urinary tract infections (AOR = 0.683, 95% CI: 0.509–0.917, *p* = 0.011) History of previous UTI (AOR = 3.209, 95% CI: 1.733–5.942, *p* = 0.001) and previous antibiotic use (AOR = 3.245, 95% CI: 1.944–5.418, *p* = 0.001) were significantly associated with UTI (Table 1).

**Table 1:** Demographic characteristics of the study participants.

Variables	Significant bacterial growth		COR (95% CI)	AOR (95% CI)	*p* value
	Growth	No growth			
Sex					
Male	87	52	1	1	
Female	149	62	1.4 (0.913–2.261)	2.5 (1.357–4.522)		0.003
Age					
10–20	19	11	1	1	
20–30	14	10				
30–40	19	5	0.98 (0.792–1.064)			0.948 (0.805–1.118)	0.528
40–50	56	30					
50–60	87	46					
60–70	29	9					
70–80	12	3					
Type of DM					
Type 1	14	12	1	1	
Type 2	222	102	1.86 (0.833–4.176)	0.582 (0.234–1.450)		0.245
Duration of DM (y)					
0–5	114	72	1	1	
5–10	59	28				
10–15	40	9					
15–20	17	5	0.665 (0.515–0.858)			0.683 (0.509–0.917)	0.011
20–25	6	0					
Drug used					
Insulin	89	47	1	1	
Metformin	147	67	0.863 (0.547–1.362)	1.160 (0.689–1.953)		0.577
Previous UTI					
Yes	170	68	1.742 (1.089–2.788)	3.209 (1.733–5.942)	0.001
No	66	46	1	1		
Catheter use					
Yes	62	30	1	1	
No	174	84	1.742 (0.600–1.658)	1.075 (0.623–1.857)		0.794
Previous antibiotic					
Yes	172	53	3.093 (1.940–4.933)	3.245 (1.944–5.418)	0.001
No	64	61	1	1		

### Prevalence of isolated uropathogens

The most common organism isolated in urine cultures was *E.coli* 220 (93.2) followed by *Klebsiella pneumoniae* 24 (10.2%), Enterococcus 17 (7.2%), 12 (5%) *Staphylococcus aureus* 6 (2.54%) *Pseudomonas aeruginosa*, 5 (2.1%) Citrobacter, and 3 (1.3%) *Proteus mirabilis*.

### Antimicrobial susceptibility testing

Different *E. coli* strains displayed different susceptibility and resistance patterns to the tested antibiotics. The tested antibiotics showed different patterns of susceptibility and resistance in the various *E. coli* strains isolated. All the uropathogenic *E. coli* strains tested were found to be resistant to cefotaxime (100%). The most effective antibiotics were found to be Colistin and imipenem, with susceptibility rates of 99.2 and 88.3%, respectively. Gentamycin also showed relatively high effectiveness, with 70% of the isolates being sensitive to it. On the other hand, ofloxacin (90.8%), ciprofloxacin (77.5%), amikacin (60.8%), and ceftriaxone (58.3%) showed high resistance rates. Cefepime resistance has a significant proportion of intermediate resistance (51.7%) as shown in Tables 2, 3.

**Table 2:** Prevalence of isolated uropathogens isolated from diabetic patients.

Organism	Frequency	Percent
E.coli	220	93.2%
*Klebsiella pneumoniae*	24	10.2%
Enterococcus	17	7.2%
*Staphylococcus aureus*	12	5%
*Pseudomonas aeruginosa*	6	2.54%
Citrobacter	5	2.1%
*Proteus mirabilis*	3	1.3%

**Table 3:** Antimicrobial susceptibility pattern of *Escherichia coli* isolates from diabetic patients in Mogadishu, Somalia.

Antibiotics	Frequency (%)	
	Sensitive	Resistance
Amikacin	86 (39.1)	134 (60.9)
Gentamycin	154 (70)	66 (30)
Cefepime	106 (48.2).	114 (51.8).
Cefotaxime	0 (0)	220 (100).
Ciprofloxacin	49 (22.27)	171 (77.8)
Ofloxacin	20 (9%)	200 (91%)
Meropenem	195 (88.6%)	25 (11.4)
Colistin	219 (99.6%)	1 (0.4)

## Discussion

The current study’s primary results indicated that bacteriuria among individuals with diabetes was 67.5%. Interestingly, none of the factors examined in the study, such as patient age, duration of diabetes, a drug used for DM management, and type of diabetes, showed any association with the prevalence of urinary tract infections (UTIs). These findings align with previous research conducted on diabetic patients in Sudan (Hamdan et al., 2015). The study found that females have a significantly higher likelihood of significant bacterial growth compared to males, with an adjusted odds ratio (AOR) of 2.5 (95% CI: 1.357–4.522, *p* = 0.003). This finding aligns with previous studies reporting a higher prevalence of bacterial growth in females (Czajkowski et al., 2021). This is mainly due to the short urethra, absence of prostatic secretion, pregnancy, and easy contamination of the urinary tract with fecal flora (Demilie et al., 2012). A history of urinary tract infection (UTI) was found to be a significant factor, with individuals having a history of UTI being over three times more likely to have significant bacterial growth compared to those without a history of UTI (AOR: 3.209, 95% CI: 1.733–5.942, *p* = 0.001). This finding aligns with previous studies reporting a higher likelihood of bacterial growth in individuals with a history of UTI (Woldemariam et al., 2019) individuals with a history of antibiotic use being over three times more likely to have significant bacterial growth than those without a history of antibiotic use (AOR: 3.245, 95% CI: 1.944–5.418, *p* = 0.001). These findings are consistent with previous studies conducted in Ethiopia (Woldemariam et al., 2019).

In this study, *E. coli* was the most predominant bacterial isolate 220 (93.2%). this is higher than the previous study conducted in Mogadishu (Mohamed et al., 2020). The second most frequently isolated bacteria were *Klebsiella pneumoniae* 24 (10.2%). A similar finding has been reported in southwest, Ethiopia (Beyene and Tsegaye, 2011).

In the present study, we observed intermediate to low-level resistance in *E. coli* to one or more antimicrobial agents. Specifically, it showed resistance primarily to cefotaxime, ciprofloxacin, ofloxacin, and amikacin. These findings align with previous reports from Ethiopia (Woldemariam et al., 2019). Gentamicin was the most effective antibiotic with 70% sensitivity. Cefepime and Meropenem also showed reasonable effectiveness, with sensitivities of 48.2 and 88.6%, respectively. Resistance to ciprofloxacin and ofloxacin was notably high, with sensitivities of only 22.27 and 9%, respectively. This is concerning because fluoroquinolones, like ciprofloxacin and ofloxacin, are frequently prescribed to treat infections caused by Gram-negative bacteria, including *E. coli*. The results of our study revealed that colistin exhibited a high level of effectiveness, as only one isolate showed resistance. This is particularly noteworthy because colistin is commonly reserved as a last-resort treatment when other antibiotics have failed to be effective. Our findings are consistent with previous studies conducted in Somalia, which have found high rates of resistance to commonly used antibiotics such as ciprofloxacin and amikacin in *Escherichia coli* isolated from diabetic patients (Mohamed et al., 2020). Similarly, a study conducted in Somalia found high resistance rates to Cefotaxime and ciprofloxacin but also found that imipenem was an effective antibiotic (Mohamed et al., 2022). The high levels of resistance observed in our study suggest a need for monitoring and judicious use of antibiotics. It is also important to explore alternative treatment options, such as the use of imipenem, to address the high levels of antibiotic resistance observed in this study. When comparing our study’s findings to those of other studies conducted outside of the study area and Africa, it is clear that antimicrobial resistance in *Escherichia coli* infections is a global concern. a study conducted in India found high rates of resistance to commonly used antibiotics such as norfloxacin and ciprofloxacin (Vidya et al., 2021). Similarly, a study conducted in Zimbabwe found high rates of resistance to ciprofloxacin and Cefotaxime (Demilie et al., 2012).

## Conclusion

Our study highlights a high prevalence of antimicrobial resistance in *Escherichia coli* strains from diabetic patients. Individuals with a history of antibiotic use are over three times more likely to exhibit significant bacterial growth. There is a critical gap in public awareness regarding proper antibiotic use. Addressing this gap through education and targeted interventions is crucial to mitigate antimicrobial resistance and improve healthcare outcomes for diabetic patients.

## Data availability statement

The raw data supporting the conclusions of this article will be made available by the authors, without undue reservation.

## Ethics statement

The studies involving humans were approved by Jamhuriya University Research Ethics Committee. The studies were conducted in accordance with the local legislation and institutional requirements. The participants provided their written informed consent to participate in this study.

## Author contributions

SH: conception, original draft writing, data collection, methodology, and formal analysis. YA: data management, methodology, formal analysis, writing, review and editing, visualization, and supervision. GH: data collection, data analysis, and review and editing. All authors contributed to the article and approved the submitted version.

## Conflict of interest

The authors declare that the research was conducted in the absence of any commercial or financial relationships that could be construed as a potential conflict of interest.

## Publisher’s note

All claims expressed in this article are solely those of the authors and do not necessarily represent those of their affiliated organizations, or those of the publisher, the editors and the reviewers. Any product that may be evaluated in this article, or claim that may be made by its manufacturer, is not guaranteed or endorsed by the publisher.
